# Immune response CC chemokines CCL2 and CCL5 are associated with pulmonary sarcoidosis

**DOI:** 10.1186/1755-1536-4-10

**Published:** 2011-04-04

**Authors:** Vyacheslav Palchevskiy, Nastran Hashemi, Stephen S Weigt, Ying Ying Xue, Ariss Derhovanessian, Michael P Keane, Robert M Strieter, Michael C Fishbein, Jane C Deng, Joseph P Lynch, Robert Elashoff, John A Belperio

**Affiliations:** 1 Division of Pulmonary and Critical Care Medicine, Department of Medicine, David Geffen School of Medicine at UCLA, Los Angeles, CA, USA; 2 Department of Medicine, St Vincent's University Hospital and University College Dublin, Dublin, Ireland; 3 Division of Pulmonary and Critical Care Medicine, Department of Medicine, University of Virginia Health System, Charlottesville, VA, USA; 4 Department of Pathology and Laboratory Medicine, David Geffen School of Medicine at UCLA, Los Angeles, CA, USA; 5 Department of Biomathematics, UCLA, Los Angeles, CA, USA

## Abstract

**Background:**

Pulmonary sarcoidosis involves an intense leukocyte infiltration of the lung with the formation of non-necrotizing granulomas. CC chemokines (chemokine (C-C motif) ligand 2 (CCL2)-CCL5) are chemoattractants of mononuclear cells and act through seven transmembrane G-coupled receptors. Previous studies have demonstrated conflicting results with regard to the associations of these chemokines with sarcoidosis. In an effort to clarify previous discrepancies, we performed the largest observational study to date of CC chemokines in bronchoalveolar lavage fluid (BALF) from patients with pulmonary sarcoidosis.

**Results:**

BALF chemokine levels from 72 patients affected by pulmonary sarcoidosis were analyzed by enzyme-linked immunosorbent assay (ELISA) and compared to 8 healthy volunteers. BALF CCL3 and CCL4 levels from pulmonary sarcoidosis patients were not increased compared to controls. However, CCL2 and CCL5 levels were elevated, and subgroup analysis showed higher levels of both chemokines in all stages of pulmonary sarcoidosis. CCL2, CCL5, CC chemokine receptor type 1 (CCR1), CCR2 and CCR3 were expressed from mononuclear cells forming the lung granulomas, while CCR5 was only found on mast cells.

**Conclusions:**

These data suggest that CCL2 and CCL5 are important mediators in recruiting CCR1, CCR2, and CCR3 expressing mononuclear cells as well as CCR5-expressing mast cells during all stages of pulmonary sarcoidosis.

## Background

Sarcoidosis is an immune-mediated multisystem disease of unknown etiology [1]. It involves the lung in over 90% of the cases and is a common cause of interstitial lung disease with some patients eventually requiring lung transplantation [1-3]. Pulmonary sarcoidosis is diagnosed via compatible clinicoradiographic findings with histological evidence of non-necrotizing granulomas in the absence of infection or other causative etiologies that can also cause pulmonary granulomas [4]. Pathologically, sarcoidosis is characterized by an initial T cell alveolitis that is dominated by a type 1 immune response environment [5-7]. This response leads to the recruitment and activation of mononuclear cells that eventually evolves into non-necrotizing granulomas [4,5]. Importantly, at some point between the transition from stage I to III pulmonary sarcoidosis there may be a conversion from a local inflammatory type 1 response to a more fibroproliferative type 2 response where inflammation gives way to pulmonary fibroplasia.

Chemokines recruit mononuclear cell subpopulations via specific receptors [8]. Different types of chemokines have been implicated in early and late stages of sarcoidosis [9]. For instance, we and others have found that the type 1 immune response interferon inducible CXC chemokines (CXCL9, CXCL10, and CXCL11) are associated with the early stages (stage I and II) but not late stage III pulmonary sarcoidosis [10-12]. Thus, it is plausible that alternative chemokines may be involved in the transition from early inflammatory stages (for example, type 1 immune responses) to late profibrotic stages (for example, type 2 immune responses) of pulmonary sarcoidosis. The CC chemokine subfamily themselves (for example, chemokine (C-C motif) ligand 5 (CCL5; also known as 'regulated upon activation, normal t cell expressed, and secreted' (RANTES)), CCL3 (macrophage inflammatory protein (MIP)-1α), CCL4 (MIP-1β), CCL2 (monocyte chemotactic protein-1 (MCP-1))) have been shown to straddle the continuum from type 1 to type 2 immune responses making these chemokines likely candidates to be involved in the overall pathogeneses of pulmonary sarcoidosis [13]. There are conflicting results with regards to CC chemokines and their association with pulmonary sarcoidosis [14-22]. This study was designed to determine whether there are augmented levels of CC chemokines in bronchoalveolar lavage fluid (BALF) from patients with the clinical/pathological diagnosis of pulmonary sarcoidosis and to resolve the current discrepancies within the literature. Moreover, this study aims to elucidate the cellular sources of specific CC chemokines and their receptors during the pathogenesis of pulmonary sarcoidosis.

## Results

### Patient characteristics

We identified 72 patients affected with different stages of pulmonary sarcoidosis as well as 8 healthy volunteers (Table 1). More specifically, there were 27 (38%) patients with stage I disease, 36 (50%) with stage II disease and 9 (12%) with stage III disease (Table 1). Using the definition of active pulmonary lymphocytic alveolitis as a bronchoalveolar lavage (BAL) lymphocyte count greater than 30 × 10^3 ^lymphocytes/ml, there were 27 (37%) patients with and 45 (63%) patients without an active alveolitis (Table 1). In all, 8 (11%) patients were on and 64 (89%) patients were off empiric immunosuppressive treatment at the time of their diagnostic bronchoscopy (Table 1). Additional pulmonary sarcoidosis patient characteristics such as age, sex, race and pulmonary function testing are outlined in Tables 1 and 2. Eight healthy volunteers without pulmonary symptoms, with normal chest radiographs and no relevant medical history underwent a bronchoscopy and were deemed the healthy control group.

**Table 1:** Characteristics of the pulmonary sarcoidosis patients.

*Pulmonary Sarcoidosis Stages*	*Patients (n)*	*Age, years*	*Sex*		*Race*			*Lymphocytic Alveolitis*	*Immuno-suppressive Therapy*
					
			*Males*	*Females*	*African-Americans*	*White*	*Others*		
I	27	39 ± 8	17(63)	10(37)	9(34)	12(44)	6(22)	13(48)	5(18)

II	36	39 ± 11	15(42)	21(58)	12(34)	20(55)	4(11)	12(33)	3(8)

III	9	54 ± 10	2(22)	7(78)	3(33)	5(55)	1(12)	2(22)	0(0)

Data are presented as mean ± SD or n (%), unless otherwise stated.

**Table 2:** Pulmonary function test results of the pulmonary sarcoidosis patients.

*Pulmonary Sarcoidosis Stages*	*FEV1 L*	*FEV1% predicted*	*FVC L*	*FVC % predicted*	*TLC L*	*TLC % predicted*	*DLCO ml min-1 mHg-1*	*DLCO % predicted*
I	3.1 ± 0.9	87.6 ± 21.2	3.8 ± 1.3	85.6 ± 21.3	5.9 ± 0.9	84.4 ± 18.9	27.1 ± 6.9	90.6 ± 17.2

II	2.9 ± 1.0	82.8 ± 21.4	3.7 ± 1.1	83.5 ± 18.9	5.5 ± 1.1	83.8 ± 37.8	23.2 ± 7.4	70.2 ± 16.9

III	1.5 ± 0.6	61.7 ± 25.0	2.4 ± 1.1	73.7 ± 33.3	3.3 ± 0.0	71.0 ± 0.0	13.5 ± 2.1	61.0 ± 8.5

Data are presented as mean ± SD or n (%), unless otherwise stated. FEV1: forced expiratory volume in 1 sec; FVC: forced vital capacity; TLC: total lung capacity; DLCO: diffusing capacity of the lung for carbon monoxide.

### CCL3 and CCL4 protein levels in BALF are not elevated in pulmonary sarcoidosis

**Figure 1:**
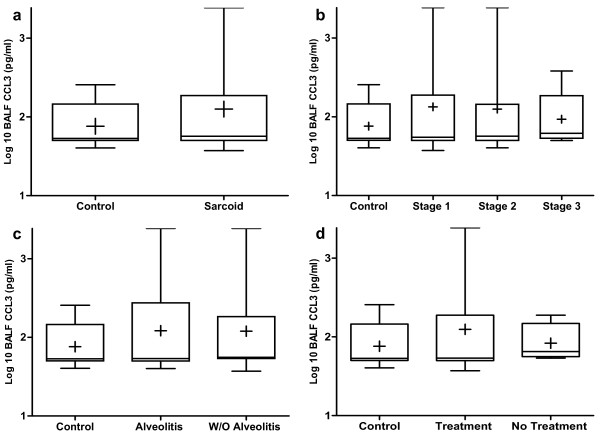
**Bronchoalveolar lavage fluid (BALF) protein levels of chemokine (C-C motif) ligand 3 (CCL3) from patients with sarcoidosis were not elevated as compared to normal healthy controls**. BALF protein levels of CCL3 by enzyme-linked immunosorbent assay (ELISA) from **(a) **all sarcoidosis patients (n = 72) compared with normal healthy volunteers (n = 8), **(b) **different stages of sarcoidosis (stage I (n = 27), stage II (n = 36) and stage III (n = 9)), **(c) **sarcoid patients with (n = 27) and without (n = 45) alveolitis, and **(d) **sarcoidosis patients with (n = 8) and without (n = 64) empirical treatment at the time of diagnosis.

**Figure 2:**
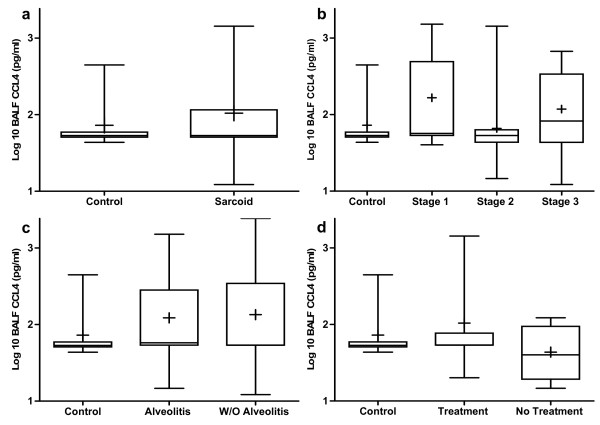
**Bronchoalveolar lavage fluid (BALF) protein levels of chemokine (C-C motif) ligand 4 (CCL4) from patients with sarcoidosis were not elevated as compared to normal healthy controls**. BALF protein levels of CCL4 by enzyme-linked immunosorbent assay (ELISA) from **(a) **all sarcoidosis patients (n = 72) compared with normal healthy volunteers (n = 8), **(b) **different stages of sarcoidosis (stage I (n = 27), stage II (n = 36) and stage III (n = 9)), **(c) **sarcoid patients with (n = 27) and without (n = 45) alveolitis, and **(d) **sarcoidosis patients with (n = 8) and without (n = 64) empirical treatment at the time of diagnosis.

BALF CCL3 and CCL4 protein levels measured by enzyme-linked immunosorbent assay (ELISA) were not significantly elevated in patients with sarcoidosis (n = 72) compared with normal healthy controls (n = 8) (Figures 1a and 2a). There were no significant differences in BALF CCL3 and CCL4 protein levels comparing healthy controls to patients with stages I, II or III sarcoidosis (Figures 1b and 2b). There were no significant differences in BALF CCL3 and CCL4 protein levels in a three-group comparison across the different stages of sarcoidosis (stage I (n = 27), stage II (n = 36) and stage III (n = 9)) (Figures 1b and 2b). The presence of a lymphocytic alveolitis (n = 27) had no impact on CCL3 and CCL4 levels (Figures 1c and 2c). Furthermore, empiric immunosuppressive therapy (n = 8) at the time of diagnostic bronchoscopy did not affect CCL3 and CCL4 protein levels (Figures 1d and 2d).

### CCL5 protein levels in BALF are elevated in pulmonary sarcoidosis and the cellular sources are mononuclear cells that form the non-necrotizing granulomas

**Figure 3:**
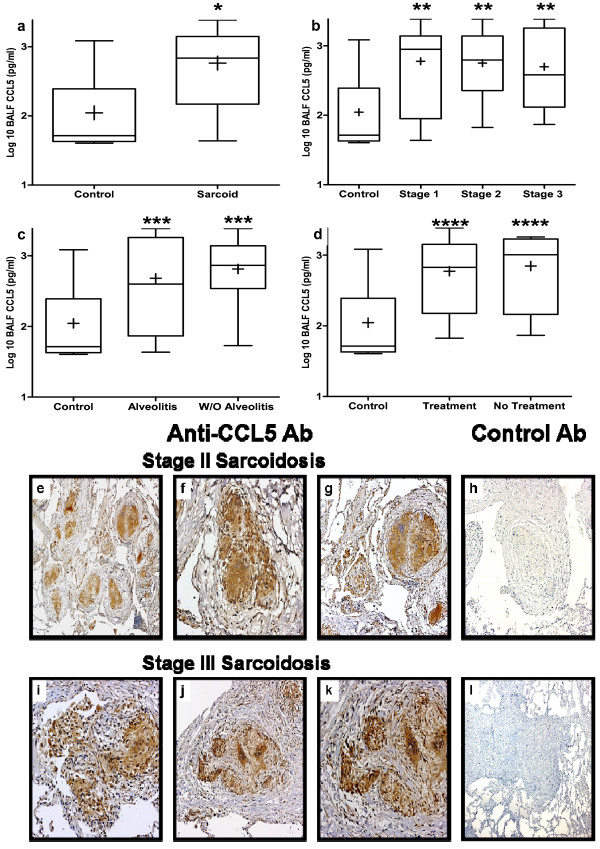
**Bronchoalveolar lavage fluid (BALF) protein levels of chemokine (C-C motif) ligand 5 (CCL5) from patients with sarcoidosis were elevated as compared to normal healthy controls**. BALF protein levels of CCL5 by enzyme-linked immunosorbent assay (ELISA) from **(a) **all sarcoidosis patients (n = 72) compared with normal healthy volunteers (n = 8), **(b) **different stages of sarcoidosis (stage I (n = 27), stage II (n = 36) and stage III (n = 9)), **(c) **sarcoid patients with (n = 27) and without (n = 45) alveolitis, and **(d) **sarcoidosis patients with (n = 8) and without (n = 64) empirical treatment at the time of diagnosis. *P = 0.002 for sarcoid versus normal; **P < 0.04 for stage I, II, and III verses normal; ***P < 0.04 for with or without alveolitis versus normal; ****P < 0.04 for with or without treatment verses normal. Representative immunohistochemistry for CCL5 **(e-g) **and isotype control antibody **(h) **on lung biopsy tissue from stages II pulmonary sarcoidosis without alveolitis and on empiric therapy. Representative immunohistochemistry for CCL5 **(i-k) **and isotype control antibody **(l) **on lung biopsy tissue from stages III pulmonary sarcoidosis without alveolitis or empiric therapy. CCL5 protein expression was found in epithelioid histiocytes and multinucleated giant cells forming non-necrotizing granulomas, as well as from surrounding alveolar macrophages and other infiltrating mononuclear cells. Panels were photographed at 50 × (e, i, h, l), 100 × (f, j), and 200 × (g, k) original magnification.

The BALF protein levels of CCL5 were significantly higher in sarcoidosis patients as compared to controls (Figure 3a). Subgroup analysis demonstrated that CCL5 levels were significantly higher in all stages (stage I, II, III), as compared to healthy controls (Figure 3b). There were no differences between patients with and without alveolitis (Figure 3c). Similarly, there were no differences between those patients who were receiving or not receiving empiric immunosuppressive therapy targeted at pulmonary sarcoidosis (Figure 3d).

Using immunohistochemical techniques we determined the cellular sources of CCL5 during all stages of pulmonary sarcoidosis, those with and without alveolitis, and those on or off empiric therapy. We found that the main cellular sources of CCL5 were epithelioid histiocytes, multinucleated giant cells, surrounding alveolar macrophages and a minor component was other inflammatory mononuclear cells that formed the non-necrotizing granulomas (Figure 3e). This pattern was consistent in all stages of sarcoidosis irrespective of alveolitis or concurrent immunosuppressive therapy, with the exception of stage III pulmonary sarcoidosis in which there were no cases that were on therapy at the time of diagnostic bronchoscopy.

### CCL2 protein levels in BALF are elevated in pulmonary sarcoidosis and the cellular sources are mononuclear cells that form the non-necrotizing granulomas

**Figure 4:**
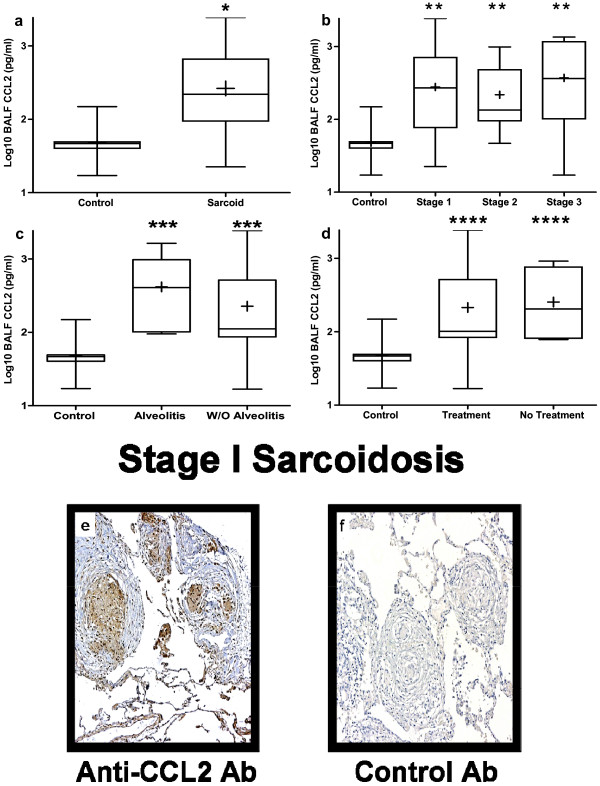
**Bronchoalveolar lavage fluid (BALF) protein levels of chemokine (C-C motif) ligand 2 (CCL2) from patients with sarcoidosis were elevated as compared to normal healthy controls**. BALF protein levels of CCL2 by enzyme-linked immunosorbent assay (ELISA) from **(a) **all sarcoidosis patients (n = 72) compared with normal healthy volunteers (n = 8), **(b) **different stages of sarcoidosis (stage I (n = 27), stage II (n = 36) and stage III (n = 9)), **(c) **sarcoid patients with (n = 27) and without (n = 45) alveolitis, and **(d) **sarcoidosis patients with (n = 8) and without (n = 64) empirical treatment at the time of diagnosis. *P = 0.001 for sarcoid versus normal; **P < 0.004 for stage I, II, and III verses normal; ***P < 0.004 for with or without alveolitis versus normal; ****P < 0.01 for with or without treatment verses normal. Representative immunohistochemistry for **(e) **CCL2 and **(f) **isotype control antibody on lung biopsy tissue from stage I pulmonary sarcoidosis without alveolitis and on empiric therapy. CCL2 protein expression was found in epithelioid histiocytes and multinucleated giant cells forming non-necrotizing granulomas, as well as from surrounding alveolar macrophages and other infiltrating mononuclear cells. Panels were photographed at 50 × original magnification.

The BALF protein levels of CCL2 were significantly higher in sarcoidosis patients as compared to controls (Figure 4a). Subgroup analysis demonstrated that BALF protein levels of CCL2 were significantly higher in all stages, as compared to controls (Figure 4b). There were no significant differences between patients with and without alveolitis or between those patients who were receiving or not receiving empiric immunosuppressive therapy targeted at pulmonary sarcoidosis (Figure 4c,d).

Using immunohistochemical techniques we determined the cellular sources of CCL2 during all stages of pulmonary sarcoidosis including those with and without alveolitis and those on or off empiric therapy. We found that the main cellular source of CCL2 was epithelioid histiocytes, multinucleated giant cells, surrounding alveolar macrophages with a minor component being other infiltrating mononuclear cells that formed the non-necrotizing granulomas (Figure 4e). This pattern was consistent in all stages of sarcoidosis irrespective of alveolitis or concurrent immunosuppressive therapy, with the exception of stage III pulmonary sarcoidosis in which there were no cases that were on therapy at the time of diagnostic bronchoscopy.

### Cellular sources of CC chemokine receptor type 1 (CCR1), CCR2, CCR3 and CCR5 during pulmonary sarcoidosis

**Figure 5:**
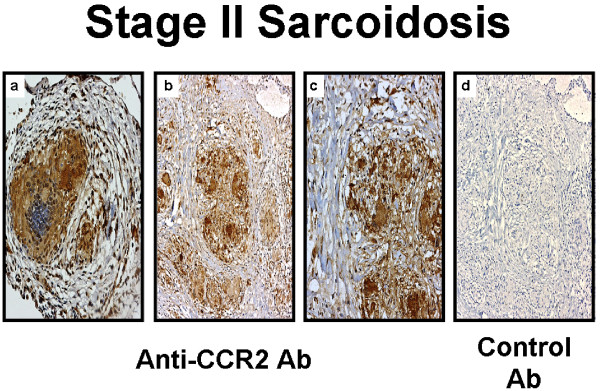
**Immunohistochemistry for CC chemokine receptor type 2 (CCR2) on lung biopsy tissues**. Representative immunohistochemistry for CCR2 **(a-c) **and isotype control antibody **(d) **on lung biopsy tissue from stages II pulmonary sarcoidosis without alveolitis and on empiric therapy. CCR2 protein expression was found in epithelioid histiocytes and multinucleated giant cells forming non-necrotizing granulomas, as well as from surrounding alveolar macrophages and other infiltrating mononuclear cells. Panels were photographed at 50 × (a, d), 100 × (b), and 200 × (c) original magnification.

**Figure 6:**
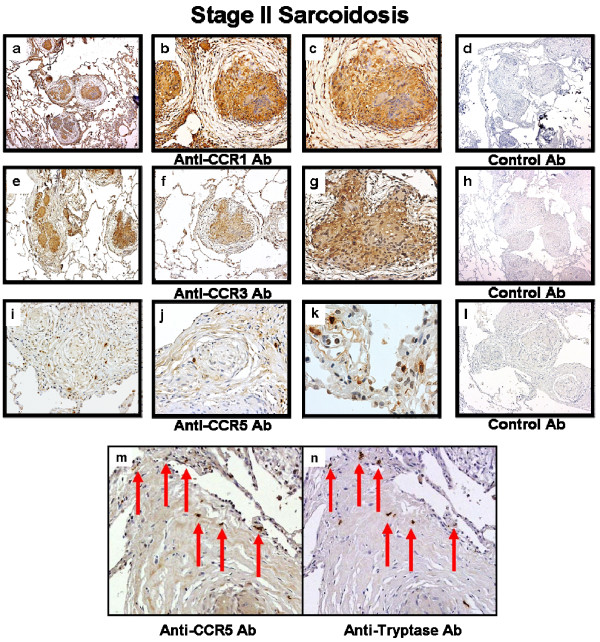
**Immunohistochemistry for CC chemokine receptor type 1 (CCR1), CCR3 and CCR5 on lung biopsy tissues**. Representative immunohistochemistry for CCR1 **(a-d)**, CCR3 **(e-g)**, CCR5 **(i-k, m)**, mast cells via tryptase **(n) **and appropriate isotype control antibodies **(d, h, l) **on lung biopsy tissue from stage II pulmonary sarcoidosis without alveolitis and on empiric therapy. CCR1 and CCR3 protein expression was found in epithelioid histiocytes and multinucleated giant cells forming non-necrotizing granulomas, as well as from surrounding alveolar macrophages and other infiltrating mononuclear cells. CCR5 protein expression was found in mast cells (i-k, m-n). The red arrows indicate mast cells (n) are expressing CCR5 (m). Panels were photographed at 50 × (a, d, e, h, i, l), 100 × (b, f, j, m, n) and 200 × (c, g, k) original magnification.

The mononuclear cell receptor(s) for CCL2 is CCR2 and for CCL5 are CCR1, CCR3 and CCR5. We found that at all stages of pulmonary sarcoidosis including those with or without alveolitis and those on or off immunosuppressive therapy main cellular sources of CCR1, CCR2, and CCR3 were epithelioid histiocytes, multinucleated giant cells and other infiltrating mononuclear cells that formed the non-necrotizing granulomas (Figure 5). However, when we evaluated the cells expressing CCR5 we found the only cell type expressing this chemokine receptor were mast cells infiltrating the non-necrotizing granulomas (Figure 6i-k, m, n). This finding was consistent irrespective of sarcoid stage, alveolitis, or concurrent immunosuppressive therapy.

## Discussion

The CC chemokine cascades involved in the recruitment of mononuclear cells that eventually leads to the formation of non-necrotizing granulomas during pulmonary sarcoidosis has not been fully elucidated. While there have been multiple studies determining if alterations in CC chemokines are associated with sarcoidosis, collectively there are many inconsistencies [14-22]. In an effort to clarify discrepancies between these studies, we performed the largest observational study involving BALF CC chemokines (CCL2, CCL3, CCL4, and CCL5) during the pathogenesis of pulmonary sarcoidosis.

CCL3 and CCL4 are CC chemokines that are known to recruit mononuclear cells with CCL3 having a preferential activity for CD8 T cells and CCL4 for CD4 T cells [23]. Two studies have found associations between elevated levels of CCL3 or CCL4 protein/cell pellet mRNA expression from BALF in patients with sarcoidosis [14,17]. Specifically, Oshima and associates using a cohort of pulmonary sarcoidosis patients (n = 17) found that the expression of CCL3 from BALF cell pellets was elevated as compared to other interstitial lung diseases (n = 10) [17]. Likewise, Capelli and colleagues demonstrated higher BALF CCL3 and CCL4 protein levels from sarcoidosis patients (n = 30) as compared to healthy controls (n = 18) [14]. In the present study, we analyzed the largest cohort of pulmonary sarcoidosis patients for CCL3 and CCL4 (n = 72) and did not find alterations in protein levels compared to healthy controls. We also performed exploratory subgroup analyses and could not demonstrate differences among different stages of sarcoidosis, those cases with or without alveolitis, or those cases on or off empiric immunosuppressive therapy at the time of diagnosis. These finding are corroborated by a smaller sarcoidosis cohort [22] and suggest that CCL3 and CCL4 are unlikely to have a significant role in the pathogenesis of pulmonary sarcoidosis.

Differences in BALF CCL5 protein levels were not found in a cohort of sarcoidosis patients (n = 22) as compared to healthy volunteers [22]. Similar results were found in a larger study of sarcoidosis patients (n = 60) [18]. Conversely, we found an association between elevated BALF protein levels of CCL5 and pulmonary sarcoidosis. Interestingly, the stage of pulmonary sarcoidosis, alveolitis, and immunosuppressive therapy at the time of pulmonary sarcoidosis diagnosis did not significantly affect the levels of CCL5. To confirm these results we evaluated the sources of CCL5 from lung biopsy tissue from all stages of pulmonary sarcoidosis, those with or without alveolitis and those on or off empiric immunosuppressive therapy. In all cases CCL5 was being expressed from epithelioid histocytes, multinucleated giant cells, surrounding alveolar macrophages and other infiltrating mononuclear cells creating the sarcoid lung granulomas. These immunohistochemistry results explain the elevated level of CCL5 found in patients with or without alveolitis and those on or off therapy for pulmonary sarcoidosis as all the non-necrotizing lung granulomas were producing CCL5 (for example, despite therapy or no alveolitis). Furthermore, we feel the reasons for the differences between our results and those from the other large study [18] has to do with the differences in study design. More specifically, we only included 'pulmonary' sarcoidosis patients (appropriate radiographic changes and lung biopsies with non-necrotizing granulomas) whereas the other study included BALF from patients with stage 0 sarcoidosis (no radiographic feature and no evidence of non-necrotizing granulomas in lung biopsies). Thus, the BALF protein level of CCL5 in those patients with stage 0 sarcoidosis would expectantly have no alterations as there are no granulomatous lesions present to produce CCL5. Moreover, our results are supported by other smaller investigations that have also found BALF proteins levels of CCL5 to be associated with pulmonary sarcoidosis [16,19].

CCL5 chemotactic activity for mononuclear cells involves three CC chemokine receptors (CCR1, CCR3, and CCR5) [8,24]. We determined what the cellular sources of these receptors were in pulmonary sarcoidosis. We found that epithelioid histocytes, macrophages and infiltrating lymphocytes that form the pulmonary sarcoid non-necrotizing granuloma were all expressing CCR1 and CCR3. Surprisingly, CCR5 was only found on non-necrotizing granuloma infiltrating mast cells. This suggests that CCL5 involvement in sarcoid granulomatous formation is important for mononuclear phagocyte, lymphocyte as well as mast cell recruitment. There are reports suggesting the involvement of mast cells in pulmonary sarcoidosis [25-27] as well as other fibrotic lung disorders [28-30]. Collectively, with our study demonstrating that CCL5 is altered in all stages of sarcoidosis it is conceivable that CCL5 via its recruitment effect on mononuclear cells, including mast cells, could be a key chemokine that is involved in the continuum of intense inflammatory injury during early stages to the more chronic inflammatory/fibrosis during late stages of pulmonary sarcoidosis.

Multiple studies involving either BALF protein levels or cell pellet mRNA expression of CCL2 have found no significant association between CCL2 and sarcoidosis [16,17]. To the contrary, our results demonstrated marked elevations of BALF protein levels of CCL2 in patients with pulmonary sarcoidosis. The levels of CCL2 were elevated in every stage of pulmonary sarcoidosis, in those with or without alveolitis and in those on or off immunosuppressive therapy. These findings were substantiated by immunohistochemical studies demonstrating CCL2 expression by epithelioid histiocytes, multinucleated giant cells within granulomas, as well as surrounding alveolar macrophages in all stages of sarcoidosis. These results are supported by smaller studies demonstrating increased protein levels or mRNA expression of CCL2 in BALF from pulmonary sarcoidosis patients [15,18,20,21]. Interestingly, we found that CCR2, the only receptor for CCL2, was expressed by the same cell types that express CCL2. Previous investigations found that CCL2 can augment cellular expression of transforming growth factor β and lead to fibroplasia [31]. Collectively, these results indicate that the CCR2/CCL2 biological axis may act in an autocrine and paracrine manner. Plausibly, this CCR2/CCL2 axis can recruit mononuclear cells that form and expand the granulomatous disease during early inflammatory stages of pulmonary sarcoidosis. However, persistent expression of CCL2 interacting with CCR2 can lead to the fibroplasia of late stages of pulmonary sarcoidosis.

Conflicting results have also been described when evaluating CCR2 polymorphisms associated with sarcoidosis [32-35]. The differences between CCR2 polymorphism investigations in sarcoidosis may be explained by linkage disequilibrium between CCR2 gene variants and polymorphisms in a neighboring sarcoidosis susceptibility gene. Collectively, these polymorphisms studies [32-35] combined with the results of our study would suggest that polymorphisms that cause CCR2 dysfunction may lead to a sarcoidosis phenotype that is less severe. However, other chemokine axes (for example, CCL5/receptors and interferon inducible ELR-CXC chemokines) may also have to be dysfunctional for these sarcoidosis patients to have significantly less severe disease.

## Conclusions

Overall this study suggests that CCL2 and CCL5 may be interacting with mononuclear cells expressing specific CC chemokines receptors (CCL2 → CCR2, CCL5 → CCR1, CCR3, and CCR5) and are important biologically in the formation of pulmonary sarcoidosis granulomas. Furthermore, unlike other chemokines (for example, interferon inducible CXC chemokines), that were only found to be elevated during early stage of pulmonary sarcoidosis [10], CCL2 and CCL5 are elevated in all stages of pulmonary sarcoidosis indicating a possible function for the continuum of early inflammatory to late fibrotic sarcoidosis. Additionally, our novel finding that CCR5 is expressed solely on the mast cells adds credence to the notion that mast cells may have some capacity to perpetuate sarcoid granulomas. Furthermore, based on our results as well as those from other investigators we think that CCL3 and CCL4 do not have a substantial role in the pathogenesis of pulmonary sarcoidosis. Only future prospective studies will be able to determine if the levels of CCL2 and CCL5 are predictive of outcomes and if the combined inhibition of these receptor(s)/ligand(s) interactions may lead to an inhibition of progression or even reversal of this lung disease.

## Methods

### Study design and patient population

With Institutional Review Board (IRB) approval and informed written consent, we prospectively enrolled all patients undergoing bronchoscopy for interstitial lung disease from June 1993 to December 2003. Patients were eligible for enrollment if they were suspected of having an interstitial lung disease. For this specific study we retrospectively identified 72 patients who had an initial de novo confirmed clinical/pathological diagnosis of pulmonary sarcoidosis. All patients underwent a bronchoscopy with transbronchial lung biopsy with or without a video-assisted thoracoscopic (VATS) biopsy, open lung biopsy or mediastinoscopy. The diagnosis of sarcoidosis was made if the patient's lung biopsy (transbronchial biopsy (n = 59, 82%) and surgical biopsy (n = 13, 18%)) showed discrete, well formed, non-necrotizing granulomas without evidence of infection or inorganic material to account for the pulmonary granulomatous reaction. Throughout the entire study we excluded any BAL performed at a time when infection and/or colonization was diagnosed via the following criteria: BALF or lung biopsy positive Gram stain or culture for bacteria, acid-fast bacillus, cytomegalovirus, respiratory viruses, *Pneumocystis jiroveci *or other fungal organisms. Additionally, patients with positive histoplasmosis serum serology or urine antigen or positive coccidioidimycoses serum serology were also excluded.

All patients with sarcoidosis were staged retrospectively using a modification of the system outlined by the American Thoracic Society (ATS)/European Respiratory Society (ERS)/World Association of Sarcoidosis and Other Granulomatous Disorders joint statement as previously described [36,37]. Criteria were as follows. Stage I: bilateral hilar lymphadenopathy without pulmonary infiltrates; stage II: bilateral hilar lymphadenopathy with pulmonary infiltrates; and stage III: pulmonary infiltrates and/or fibrosis in the absence of hilar lymphadenopathy [36,37]. In addition, we also retrospectively characterized these patients with or without an active pulmonary lymphocytic alveolitis defined by a BAL lymphocyte count greater than 30 × 10^3 ^lymphocytes/ml as previously described [10,38]. Furthermore, we identified eight sarcoid patients being treated with empiric methylprednisolone therapy for symptoms of arthralgia at the time of their diagnostic bronchoscopy. BALF was also obtained from eight healthy control patients with no relevant medical history, pulmonary symptoms or abnormal radiographical findings.

### BALF collection and process

Bronchoalveolar lavage was performed in both pulmonary sarcoidosis patients and controls using standard techniques as previously described [10]. Briefly, four 60 ml aliquots of sterile isotonic saline solutions were instilled via a flexible bronchoscope wedged into a subsegmental bronchus of a predetermined region of interest based on radiographical findings, and lavage fluid was aspirated back each time by low suction. The pooled BALF was split equally into two samples. One sample was dispatched for clinical microbiology/cytology and the other sample was placed on ice and transported to the research laboratory. The research sample was filtered through sterile gauze and centrifuged (2000 rpm for 10 min) and the supernatant fluid was immediately frozen at -70°C until thawed for chemokine analysis.

### Immunoassay

The levels of CCL2, CCL3, CCL4, and CCL5 in BALF were quantified via the manufacturer's protocol using ELISA kits (R&D Systems; Minneapolis, MN, USA).

### Immunohistochemistry for CC chemokines and their receptors

Using immunohistochemical techniques we determined the cellular sources of specific CC chemokines and their appropriate receptors during all stages of pulmonary sarcoidosis, those with and without alveolitis and those on or off empiric therapy (stage I with alveolitis and off therapy (n = 3), without alveolitis and on therapy (n = 3); stage II with alveolitis and off therapy (n = 3), without alveolitis and on therapy (n = 3); and stage III with alveolitis and off therapy (n = 2), without alveolitis and off therapy (n = 2). Reagents for immunohistochemistry included mouse anti-human CCL2, CCR1, and CCR5 monoclonal antibodies (Abs), goat anti-human CCL5 antibody, and rat anti-human CCR3 antibody, which were purchased from R&D Systems. Rabbit monoclonal anti-human CCR2 was purchased from Epitomics (Burlingame, CA, USA). Mouse monoclonal anti-human to mast cell tryptase was purchased from Abcam (Cambridge, MA, USA). The expression of CCL2 and CCL5 and their receptors CCR1, CCR2, CCR3, CCR5 as well as Tryptase was assessed by immunocytochemistry on paraffin-embedded samples of lung tissue from sarcoidosis patients as previously described [10]. Briefly, staining was performed using the VECTASTAIN ABC Standard kits (Vector Laboratories, Inc., Burlingame, CA, USA). After deparaffinization and antigen retrieval in sodium citrate buffer (pH 6.0), endogenous perioxidase was quenched with 3% hydrogen peroxide in PBS for 10 min. Non-specific binding was minimized by incubation in appropriate blocking serum for 30 min and endogenous biotin was blocked with an avidin/biotin blocking kit (Vector). Slides were then incubated overnight with primary antibodies at 4°C. Specific labeling was detected with a biotinylated specific secondary antibody and application of horseradish peroxidase-conjugated avidin-biotin followed by development with DAB solution (Vector).

### Statistical analysis

Demographic data as well as pulmonary function test values were reported as mean ± standard error of the mean (SEM). Chemokine levels of BALF had skewed distributions; therefore, we transformed all data on log 10 for analysis. Levels of CCL2, CCL3, CCL4 and CCL5 in BALF were compared between sarcoid patients and controls using an unpaired *t *test. To evaluate differences by the stages of sarcoidosis, subgroup analysis of variance (ANOVA) was performed and each subgroup was compared with controls using an unpaired *t *test. To assess the role the CC chemokines in sarcoid patients with and without alveolitis, we categorized patients into two groups based upon BAL lymphocyte counts: alveolitis >30 × 10^3 ^cells/ml and without alveolitis ≤30 × 10^3 ^cells/ml. We also performed further subgroup analysis in patients who were or were not on empiric immunosuppressive therapy at the time of pulmonary sarcoidosis diagnosis. Unpaired *t *test was used to determine the differences in those groups. Box and whisker plots were used to show the distribution of data around the mean, median, 25th and 75th interquartile ranges. All analysis was performed with GraphPad Prism 5 statistical package (GraphPad Software Inc, San Diego, CA, USA).

## Competing interests

The authors declare that they have no competing interests.

## Authors' contributions

VP, NH, YYX, MPK, JAB participated in research design, in the performance of the research, data analysis and in drafting of the manuscript. SSW, AD, RE participated in the design of the study and performed statistical analysis. RMS, MCF, JCD and JPL participated in the design of the study and helped to draft the manuscript. All authors read and approved the final manuscript.
